# Identification of IgE-binding proteins in buckwheat

**DOI:** 10.1186/2045-7022-4-S2-P13

**Published:** 2014-03-17

**Authors:** Rie Satoh, Rika Nakamura, Mayumi Ohnishi-Kameyama, Reiko Teshima

**Affiliations:** 1 NARO Food Research Institute, Analytical Science Division, Tsukuba, Ibaraki, Japan; 2 National Institute of Health Sciences, Division of Novel Foods and Immunochemistry, Tokyo, Japan; 3 National Institute of Health Sciences, Division of Foods, Tokyo, Japan

## Background

Buckwheat (*Fagopyrum esculentum*) is not only common pseudo-cereal in Japan, Korea, and other East Asian countries, but also a health food and substitute for wheat flour in Western countries. Buckwheat allergy is an immunoglobulin E (IgE)-mediated hypersensitivity manifesting as severe and critical symptoms induced by ingestion or inhalation of even a small amount of the flour or food products. It is therefore important to identify buckwheat IgE-binding proteins and clarify the mechanism of buckwheat allergy for developing an accurate diagnostic procedure and safer immunotherapy.

## Methods

The comprehensive IgE-binding proteins in buckwheat were examined using immunoproteomic techniques. Salt-soluble proteins were extracted from buckwheat seeds and seedlings, separated using one- and two-dimensional electrophoresis, and analyzed using western blotting with buckwheat-allergic patients' sera or rabbit polyclonal antibody specific to buckwheat allergen.

## Results

Immunoproteomic analysis revealed multiple IgE-binding proteins containing known or putative allergens in buckwheat. Some spots were identified as 13S globulin protein subunits or isoforms. Some spots that were homologous to vicilin-like proteins indicated the presence of newly identified vicilin-like proteins in buckwheat.

## Conclusion

The results obtained from an immunoproteomic analysis may contribute to the construction of a comprehensive IgE-binding protein map of buckwheat and the detection of isoforms of IgE-binding proteins in buckwheat variants.

